# Effect of deworming on school-aged children’s physical fitness, cognition and clinical parameters in a malaria-helminth co-endemic area of Côte d’Ivoire

**DOI:** 10.1186/1471-2334-14-411

**Published:** 2014-07-25

**Authors:** Eveline Hürlimann, Clarisse A Houngbedji, Prisca B N’Dri, Dominique Bänninger, Jean T Coulibaly, Peiling Yap, Kigbafori D Silué, Eliézer K N’Goran, Giovanna Raso, Jürg Utzinger

**Affiliations:** Department of Epidemiology and Public Health, Swiss Tropical and Public Health Institute, Basel, Switzerland; University of Basel, Basel, Switzerland; Département Environnement et Santé, Centre Suisse de Recherches Scientifiques en Côte d’Ivoire, Abidjan, Côte d’Ivoire; Unité de Formation et de Recherche Sciences de la Nature, Université Nangui Abrogoua, Abidjan, Côte d’Ivoire; Unité de Formation et de Recherche Biosciences, Université Félix Houphouët-Boigny, Abidjan, Côte d’Ivoire

**Keywords:** Anaemia, Cognition, Co-infection, Côte d’Ivoire, Deworming, Helminth, Malaria, Malnutrition, Physical fitness

## Abstract

**Background:**

Malaria and helminth infections are thought to negatively affect children’s nutritional status and to impair their physical and cognitive development. Yet, the current evidence-base is weak. The purpose of this study was to determine the effect of deworming against soil-transmitted helminthiasis and schistosomiasis on children’s physical fitness, cognition and clinical parameters in a malaria-helminth co-endemic setting of Côte d’Ivoire.

**Methods:**

We designed an intervention study with a 5-month follow-up among schoolchildren aged 5–14 years from Niablé, eastern Côte d’Ivoire. In late 2012, a baseline cross-sectional survey was conducted. Finger-prick blood, stool and urine samples were subjected to standardised, quality-controlled techniques for the diagnosis of *Plasmodium* spp., *Schistosoma* spp., soil-transmitted helminths and intestinal protozoa infections. Haemoglobin level was determined and anthropometric measurements were taken for appraisal of anaemia and nutritional status. Children underwent memory (digit span) and attention (code transmission) cognitive testing, and their physical fitness and strength were determined (20 m shuttle run, standing broad jump and grip strength test). All children were treated with albendazole (against soil-transmitted helminthiasis) and praziquantel (against schistosomiasis) after the baseline cross-sectional survey and again 2 months later. Five months after the initial deworming, the same battery of clinical, cognitive and physical fitness tests was performed on the same children.

**Results:**

Lower scores in strength tests were significantly associated with children with harbouring nutritional deficiencies. Surprisingly, boys infected with *Schistosoma mansoni* achieved longer jumping distances than their non-infected counterparts. Light-intensity infection with *S. mansoni* was associated with slightly better aerobic capacity. Deworming showed no effect on haemoglobin levels and anaemia, but children with moderate- to heavy-intensity *Schistosoma* infection at baseline gained weight more pronouncedly than non-infected children. Interestingly, children with soil-transmitted helminth or *Schistosoma* infection at baseline performed significantly better in the sustained attention test than their non-infected counterparts at the 5-month follow-up.

**Conclusions:**

This study revealed conflicting results regarding clinical parameters and cognitive behaviour of children after two rounds of deworming. We speculate that potential beneficial effects of deworming are likely to be undermined in areas where malaria is co-endemic and nutritional deficiencies are widespread.

**Electronic supplementary material:**

The online version of this article (doi:10.1186/1471-2334-14-411) contains supplementary material, which is available to authorized users.

## Background

In developing countries, preschool-aged children are at high risk of malaria, whilst school-aged children carry the highest burden of helminth infections, such as soil-transmitted helminthiasis and schistosomiasis [1–4]. Consequences of malaria and helminthiases are manifold, including clinical but also more subtle morbidities. Anaemia and malnutrition are common clinical manifestations and negatively affect childhood mental and physical development [4–6]. In 2010, malaria and the neglected tropical diseases (NTDs), of which helminth infections are of particular importance in terms of number of infections and global burden [7, 8], accounted for an estimated 6.4 million disability-adjusted life years (DALYs) among the school-aged population in sub-Saharan Africa, representing 16.5% of the total DALYs [9]. These burden estimates result from a complex construct to quantify the comparative magnitude of health loss due to diseases, injuries and risk factors. Efforts have been made for improving the assessment of the ‘true’ burden of disease by incorporating different sequelae to capture direct consequences of disease (e.g. anaemia due to hookworm infection) [10]. However, these sequelae mainly describe clinical conditions and do not sufficiently take into account subtle morbidities [11, 12].

Recent studies assessed not only clinical consequences of parasitic infections, but also included measurements on physical functioning [13–15], school performance and cognitive ability of children [16, 17]. Yap and colleagues, for example, showed that *Trichuris trichiura*-infected children in the People’s Republic of China had significantly lower age-adjusted aerobic capacity (VO_2_ max, expressed in ml kg^−1^ min^−1^) than their non-infected counterparts, as assessed by a multi-stage 20 m shuttle run test [15]. In Côte d’Ivoire, where polyparasitism is still widespread [2, 18, 19], there was no clear association between children’s infection with *Schistosoma* and soil-transmitted helminths and physical fitness [14]. *Plasmodium falciparum* infection was found to be associated with lower performance in abstract reasoning and sustained attention in Ugandan schoolchildren [17]. Ezeamama and colleagues showed that several helminth species (i.e. *Schistosoma japonicum*, *Ascaris lumbricoides* and *T. trichiura*) were negatively associated with children’s cognitive test scores in a study done in the Philippines [20].

An important limitation of most previous studies that examined children’s physical functioning and cognition in relation to infectious diseases is that the studies pursued a cross-sectional epidemiological design with no detailed follow-up investigations after an intervention. In a recent randomised controlled trial carried out with school-aged children in the People’s Republic of China, no significant improvement in physical fitness was observed 6 months after administration of a triple-dose of albendazole against soil-transmitted helminthiasis [21]. Potential explanations of the lack of a beneficial effect of deworming were the very low cure rates against *T. trichiura* and the high re-infection rates for all soil-transmitted helminth species [22]. Although some attention has been given on the effects of deworming on school performance, the current evidence-base is weak, with no obvious or consistent effect, as revealed by a systematic review and meta-analysis [23].

The purpose of this study was to assess the dynamics of children’s physical fitness, cognitive ability and clinical morbidities over a 5-month period after two rounds of deworming. The study was carried out in a malaria-helminth co-endemic area in the eastern part of Côte d’Ivoire.

## Methods

### Ethics statement

The study protocol was approved by the institutional research commissions of the Swiss Tropical and Public Health Institute (Basel, Switzerland) and the Centre Suisse de Recherches Scientifiques en Côte d’Ivoire (Abidjan, Côte d’Ivoire). Ethical approval was obtained from the ethics committees in Basel (EKBB; reference no. 30/11) and Côte d’Ivoire (CNER; reference no.: 09-2011/MSHP/CNER-P). Our study is registered at Current Controlled Trials (identifier: ISRCTN37143632).

District and village education and health authorities, parents/guardians and schoolchildren were informed about the objectives, procedures and potential risks and benefits of the study. Written informed consent was sought from children’s parents/guardians. It was emphasised that participation was voluntary and that children could withdraw anytime without further obligation. All data records were coded and kept confidential. Medical staff performed clinical examinations, supervised physical fitness tests and administered anthelminthic drugs. Children were treated twice with albendazole (400 mg) against soil-transmitted helminthiasis and praziquantel (40 mg/kg) against schistosomiasis at baseline and a 2-month follow-up survey [24]. At the end of the study, helminth-positive children were again treated with the aforementioned drugs. Children with clinical malaria (i.e. positive rapid diagnostic test (RDT) and tympanic temperature ≥38.0°C) were given artemisinin-based combination therapy (i.e. artesunate-amodiaquine) and paracetamol against fever. An anti-anaemic treatment for children with haemoglobin (Hb) levels below 100 g/l was provided in cases where no signs of clinical malaria were present.

### Study design and sample size calculation

We designed a 5-month longitudinal study. In December 2012, a baseline cross-sectional survey was conducted to determine children’s parasitological, clinical, cognitive and physical fitness status. Children were systematically administered albendazole and praziquantel; after the baseline survey and 2 months later. In May 2013, children were re-examined with the same battery of tests as in the baseline cross-sectional survey.

For sample size calculation, we considered the arithmetic mean and variance of physical fitness, as determined by VO_2_ max, in a previous study in a rural setting of south Côte d’Ivoire [14]. We assumed that a difference of 5% in VO_2_ max is of clinical relevance. Results from a recent cross-sectional survey in the current study area revealed a helminth infection prevalence of about 50% [25]. To achieve a power of 90% at an alpha error of 5% to obtain a statistical significance in VO_2_ max, and allowing for 30% drop-outs for non-compliance and incomplete follow-up assessment, we calculated that 194 children would need to be enrolled [26]. Additionally, we accounted for unexpected difference in group sizes, which call for larger sample sizes [27]. Hence, we aimed for a total of 300 children to participate.

### Study area and subjects

The study was conducted in two adjacent primary schools in the village of Niablé (geographical coordinates: 6°39’48.0” N latitude, 3°16’25.1” W longitude). Niablé is located in the Indénie-Djuablin region of eastern Côte d’Ivoire, at the border to Ghana. The village is characterised by a Guinean bio-climate with yearly average precipitation ranging between 1,200 mm and 1,700 mm [28]. The rainy season lasts from March to December and is interrupted by a short dry season in August [29]. People are mainly engaged in subsistence farming, while coffee and cocoa serve as cash crops. There are myriad stagnant water bodies (e.g. small multi-purpose dams and fish ponds within and surrounding the village, and a poorly maintained drainage system and open waste disposal sites) that foster *in situ* transmission of malaria and schistosomiasis. Niablé was selected based on a 23.6% prevalence of *Schistosoma mansoni* infection among school-aged children identified during a national cross-sectional survey conducted in November 2011 [25]. To achieve the intended sample size of 300 children aged 8–14 years, all children attending grades 4–6 in the two primary schools were invited to participate.

### Field and laboratory procedures

For parasitological examination, each child was asked to provide a fresh urine and stool sample and a finger-prick blood sample. Urine and stool samples were collected in separate plastic containers distributed to children in the early morning hours. Sample collection was done between 10:00 and 12:00 hours. Finger-prick samples were subjected to an RDT (ICT ML01 Malaria Pf kit; ICT Diagnostics, Cape Town, South Africa). Thick and thin blood films were prepared on microscope slides and air-dried.

All biological samples were transferred to a nearby laboratory and processed as follows. A small portion of stool (1–2 g) was put in Falcon tubes, filled with 10 ml of sodium acetate-acetic acid-formalin (SAF) [30]. Duplicate Kato-Katz thick smears were prepared from each stool sample, using 41.7 mg templates [31]. Urine samples were subjected to a filtration method [32] for detection of *S. haematobium* eggs. Kato-Katz thick smears and filter slides were examined under a microscope by experienced laboratory technicians. The number of helminth eggs was counted and recorded for each species separately. Thick and thin blood films were stained with a 10% Giemsa solution and examined under a microscope for *Plasmodium* species identification and quantification of parasitaemia (parasites/μl of blood) [33]. SAF-fixed stool samples were subjected to an ether-concentration technique and examined under a microscope for intestinal protozoa [34]. Helminth eggs were also recorded. Ten percent of all slides were randomly selected and re-examined by a senior microscopist for quality control.

Clinical examination was conducted by experienced medical staff. It included palpation for liver and spleen enlargement, Hb measurement using a HemoCue analyser (Hemocue Hb 301 system; Angelholm, Sweden) to assess anaemia, and measurement of body temperature using an ear thermometer (Braun ThermoScan IRT 4520; Kronberg, Germany) to identify fever cases (≥38.0°C). Anthropometric measurements such as height (to the nearest cm) and body weight (to the nearest 0.5 kg) were recorded to determine nutritional status.

The same day children provided biological samples and were examined clinically, they were invited for a questionnaire interview. Questions pertaining to household asset ownership, adapted from instruments previously used elsewhere in Côte d’Ivoire, were employed for calculating socioeconomic status [35, 36].

### Physical fitness testing

To assess children’s physical fitness, three tests from the Eurofit physical fitness test battery were employed [37]; namely (i) the standing broad jump; (ii) the grip strength; and (iii) the 20 m shuttle run tests. The first two tests measure strength, whereas the 20 m shuttle run test is designed to assess the aerobic capacity and cardio-respiratory endurance [38]. Test procedures were explained and demonstrated to the children before the actual test was conducted. The 20 m shuttle run test was executed on a flat ground without vegetation cover on the school yard either in the morning (between 8:00 and 10:00 hours) or in the late afternoon (between 16:00 and 18:00 hours) to avoid high ambient air temperature. Detailed instructions on how the tests were implemented are given as supporting information (see Additional file 1). Children identified with health problems by medical staff (e.g. Hb <80 g/l, clinical malaria or respiratory tract infection) were excluded from physical fitness testing.

### Cognitive function testing

Two cognitive tests were chosen; one focussing on sustained attention and the other on working memory. The tests were first explained in class and specific training sessions were conducted for a deeper understanding of the tasks to be performed. The code transmission test is part of the ‘Tests of Everyday Attention for Children’ (TEA-Ch) [39] and has been used before in Africa [16, 17]. During the test, a list of digits was read out aloud with a speed of one digit/sec to the child. The task of the child was to identify a ‘code’ of two consecutive 5’s, to interrupt the tester and to indicate the number preceding this code. Before the actual start of the test, each child had the possibility to familiarise with the test through four warm-up digit sequences. Those children who did not understand the principle of the task during the warm-up period were invited for a more basic attention test; the pencil tapping test [40]. Additionally, the forward digit span test, a subtest of the ‘Wechsler Intelligence Scale for Children - Fourth Edition’ (WISC-IV) [41] was conducted to assess children’s working memory. Children were asked to correctly recall and repeat a sequence of digits of increasing length in the given order.

### Statistical analysis

Data were double-entered and cross-checked using EpiInfo version 3.5.3 (Centers for Disease Control and Prevention; Atlanta, USA). Statistical analyses were performed in STATA version 10.1 (STATA Corp.; College Station, USA). For the main analysis, a two-sample strategy was applied. Data from participants with complete baseline records served as the first sample, while the second sample consisted of those children who additionally had complete data records at the 5-month follow-up survey. Data from code transmission testing were analysed only for those children who fully understood the test, while treatment efficacy was evaluated for individuals from sample 2 with complete stool and urine examinations. Children with incomplete follow-up records were excluded from the second sample and were subjected to an attrition analysis.

Helminth infections were classified into light, moderate and heavy intensity categories, according to WHO guidelines [4]. Anaemia was determined using the cut-offs of Hb <115 g/l for children aged 5–11 years and Hb <120 g/l for children aged 12–15 years [42]. *Plasmodium* spp. parasitaemia was transformed and expressed as log (parasitaemia +1) to normalise the distribution for subsequent descriptive statistics and comparison of means. Nutritional status was calculated using STATA macros from WHO child growth standards for children aged 5–19 years [43]. Indicators for malnutrition included stunting (height-for-age), wasting (body mass index (BMI)-for-age) and underweight (weight-for-age), whereof the latter was used as a valid reference measure for malnutrition in children younger than 10 years of age only.

Socioeconomic status was calculated using a household asset-based approach. Subsequently, children were stratified according to their asset index into three economic groups (wealth tertiles; e.g. most poor, poor and least poor) as done elsewhere [44]. VO_2_ max was used as the main outcome measure from the 20 m shuttle run test and was calculated for each child according to an equation provided by Léger and colleagues [38].

Chi-square and Fisher’s exact test, as appropriate, were applied for comparison of infection, morbidity and low performance in digit span test proportions between different groups in the main analysis and the attrition analysis. McNemar’s test was applied for comparison of baseline *vs*. follow-up differences. To compare continuous outcomes such as fitness scores, weight gain, *Plasmodium* parasitaemia and mean differences in cognition scores between baseline and follow-up by group, *t*-test statistics, Wilcoxon signed rank sum, one-way ANOVA and Kruskal-Wallis tests were used, respectively. A binary variable for low performance in the digit span test was defined using a cut-off for the longest spans forward (LSF) raw score, at the level of LSF ≤4 according to Iverson and Tulsky [45]. Depending on whether outcome variables were continuous, binary or overdispersed and censored count data, bivariate and multivariate linear, logistic, negative binomial and tobit regression models were utilised, as appropriate, to assess relationships with covariates. A population-averaged generalised estimating equation (GEE) and a random effects tobit regression model approach was adopted for analysis of the repeated outcome measurements of individuals with complete baseline and follow-up records. Sociodemographic, baseline parasite infection and morbidity variables served as explanatories in the regression analysis on baseline outcomes and changes after the 5-month follow-up. In addition to the covariates mentioned above, dynamic explanatories (i.e. change in anaemia status, nutritional status or *P. falciparum* parasitaemia) were introduced in the GEE and random effects models to assess associations with changes over time in the outcome measures. All cross-sectional and longitudinal models were fitted following a stepwise elimination process, excluding explanatory variables at significance level of 0.2 and considering the Akaike information criterion (AIC) and quasi-likelihood information criterion (QIC), respectively. Relationships between an outcome and explanatory variables were expressed as adjusted odds ratios (ORs) in case of logistic regression, incidence rate ratios (IRRs) for negative binomial and mean differences for linear or tobit models, respectively, with corresponding 95% confidence intervals (CIs). Presented results from GEE analysis focused on main effects (time trend) and interaction terms with time to highlight within-subject effects between groups.

Treatment efficacy 3 months after the second round of deworming was assessed by calculating cure rate (CR, defined as the proportion of baseline helminth-positive children who became egg-negative after treatment) and egg reduction rate (ERR; formula: 1 – [egg counts after treatment/egg counts at baseline] × 100 based on population geometric mean eggs counts).

## Results

### Compliance and study samples

**Figure 1:**
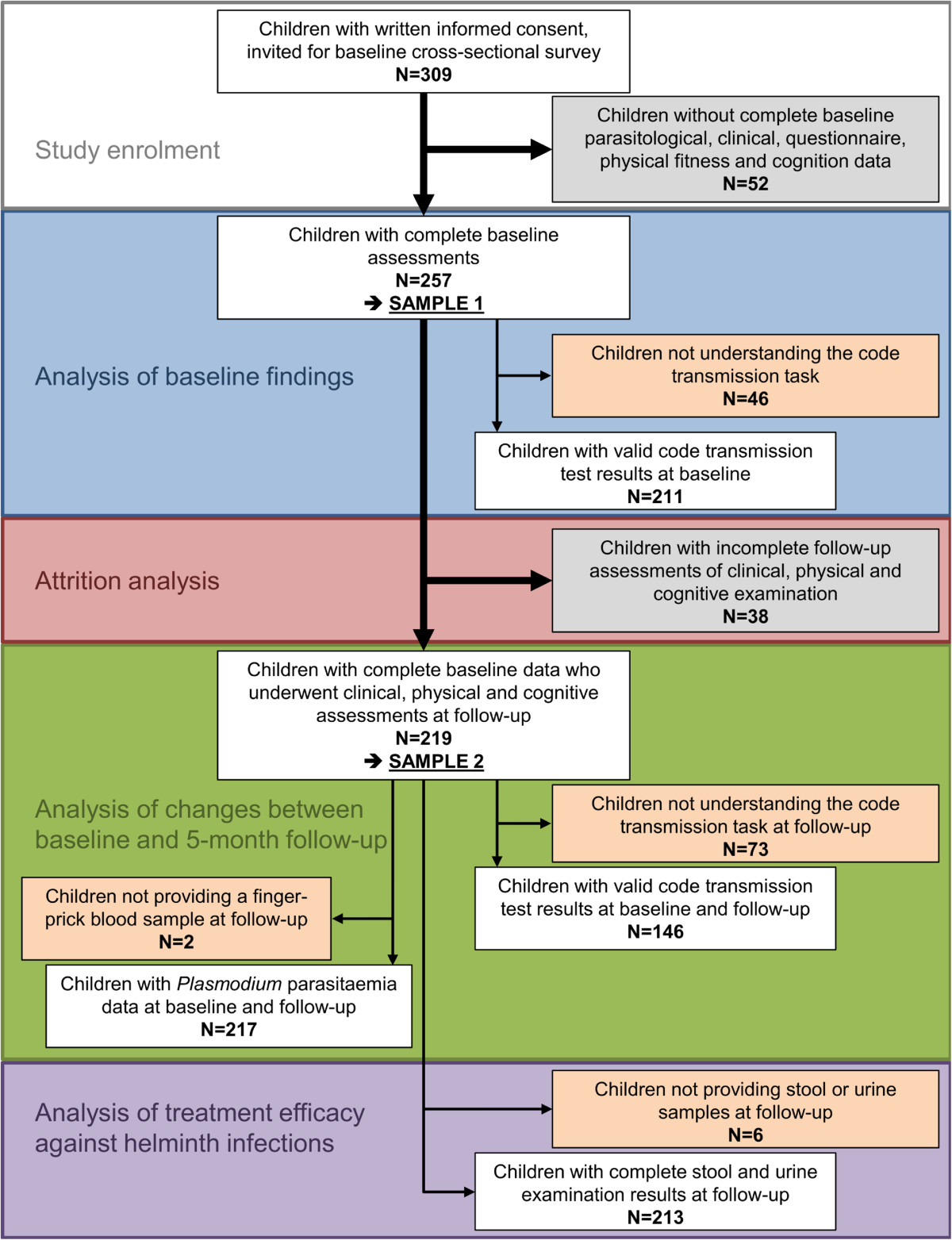
**Flow chart illustrating study participation**, **compliance and respective analysis grouping.** The baseline cross-sectional survey was carried out in December 2012, while the end-of-study survey took place in May 2013 in the village of Niablé, eastern part of Côte d’Ivoire.

Figure 1 gives a flow chart, summarising study participation. In brief, study sample 1 consisted of 257 children (134 girls; 123 boys) who had complete baseline data. Among them, 219 children (112 girls, 107 boys) also had complete clinical, physical fitness and cognitive data at the 5-month follow-up assessment, and hence served as sample 2. The mean age was 10.6 and 10.7 years in sample 1 and 2, respectively, with a range of 5–14 years. The main reason why children were excluded from sample 2 is that they missed at least one of the follow-up assessments (clinical examination, physical fitness and cognition; n = 38). 213 children from sample 2 provided stool samples at the end of the study for treatment efficacy evaluation against helminth infections, whilst 217 provided finger-prick blood samples at follow-up and were considered for analysis of changes of *Plasmodium* parasitaemia. Children not understanding the code transmission test were kept in the two samples, but only 146 children with valid results at baseline and the 5-month follow-up were considered for evaluation of dynamics in test scores.

### Results from attrition analysis

The attrition analysis of the 38 children excluded from sample 2 revealed no statistically significant differences in terms of age, sex and socioeconomic status, baseline prevalence of parasitic infections and clinical morbidity, such as anaemia and nutritional status. Moreover, children excluded from sample 2 showed comparable baseline characteristics of physical fitness and cognition than their non-excluded counterparts. Mean *Plasmodium* parasitaemia at baseline was the only parameter with a statistically significant difference; mean parasitaemia 1,872 parasites/μl of blood in the excluded children compared to 1,031 parasites/μl of blood in sample 2 (p = 0.022).

### Baseline situation

Baseline characteristics of study sample 1, including parasitic infections and clinical signs, are summarised in Table 1. *P. falciparum* was the predominant species (91.1%). *S. mansoni* was the most prevalent helminth species (35.4%), followed by hookworm (9.7%). With regard to intestinal protozoa, *Giardia intestinalis* was the predominant pathogenic species with a prevalence of 14.8%. Males were significantly more often infected with intestinal helminths, such as *S. mansoni* and hookworm. Concurrently, males were significantly more often found to be co-infected with *Plasmodium* and helminths compared to females. Anaemia was identified in 34.6% of the 257 children with a slightly higher prevalence in males than females. Boys had significantly lower Hb levels than girls. About one third of the children showed moderate or severe signs of malnutrition. Moderate to severe stunting was exclusively observed in children aged 10–14 years. There was no statistically significant (p < 0.05) difference in infection prevalence and clinical outcomes between the three wealth groups. The findings from the multivariate logistic regression analysis underline the association of clinical outcomes with age and highlight the relationship between anaemia and malnutrition (Table 2). Apart from a significant negative relationship between Hb level and *Plasmodium* parasitaemia there was no statistically significant association of clinical morbidity with parasite infections.

**Table 1:** **Baseline demographic**, **parasitological and clinical characteristics of study sample 1** (**257 schoolchildren**) **in Niablé**, **eastern Côte d**’**Ivoire in December 2012**

Characteristic	Total (n = 257)	Females (n = 134)	Males (n = 123)
Age (years)			
Mean (range)	10.6 (5–14)	10.6 (5–14)	10.7 (7–14)
Age group 5–9, no. of children (%)	65 (25.3)	37 (27.6)	28 (22.8)
Age group 10–14, no. of children (%)	192 (74.7)	97 (72.4)	95 (77.2)
School grade			
4	48 (18.7)	26 (19.4)	22 (17.9)
5	115 (44.8)	63 (47.0)	52 (42.3)
6	94 (36.6)	45 (33.6)	49 (39.8)
Infection with *P. falciparum*			
No. of children infected (%)	234 (91.1)	119 (88.8)	115 (93.5)
Parasitaemia, mean no. of parasites/μl of blood(log-transformed)	1,254 (6.2)	1,085 (6.2)	1,429 (6.2)
Infection with *P. malariae*			
No. of children infected (%)	21 (8.2)	14 (10.5)	7 (5.7)
Parasitaemia, mean no. of parasites/μl (log-transformed)	1,037 (6.5)	972 (6.6)	1,167 (6.3)
Infection with *S. mansoni*			
No. of children infected (%)**	**91** **(35.4)**	**25** **(18.7)**	**66** **(53.7)**
Infection intensity, no. of children infected (%)			
Light (1–99 EPG)	49 (53.9)	15 (60.0)	34 (51.5)
Moderate (100–399 EPG)	33 (36.3)	9 (36.0)	24 (36.4)
Heavy (≥ 400 EPG)	9 (9.9)	1 (4.0)	8 (12.1)
Infection with soil-transmitted helminths^#^			
Hookworm, no. of children infected (%)**	**25** **(9.7)**	**2** **(1.5)**	**23** **(18.7)**
* T. trichiura*, no. of children infected (%)	2 (0.8)	1 (0.8)	1 (0.8)
* A. lumbricoides*, no. of children infected (%)	1 (0.4)	1 (0.8)	0 (0.0)
Infection with pathogenic intestinal protozoa			
* G. intestinalis*, no. of children infected (%)	38 (14.8)	16 (11.9)	22 (17.9)
* E. histolytica*/*E. dispar*, no. of children infected (%)	11 (4.3)	5 (3.7)	6 (4.9)
Major co-infections			
* Plasmodium* spp. + helminth, no. of children co-infected (%)**	**97** **(37.7)**	**27** **(20.2)**	**70** **(56.9)**
* Plasmodium* spp. + pathogenic intestinal protozoa, no. of children co-infected (%)	44 (17.1)	19 (14.2)	25 (20.3)
* P. falciparum* + *S. mansoni*, no. of children co-infected (%)**	**86** **(33.5)**	**23** **(17.2)**	**63** **(51.2)**
Haemoglobin levels and anaemia			
Hb, mean (SD), g/l*	**120.2** **(13.6)**	**122.3** **(13.0)**	**118.0** **(13.9)**
Anaemia, no. of children (%)	89 (34.6)	40 (29.9)	49 (39.8)
Malnutrition			
Any form of malnutrition (Z ≤ −2), no. of children (%)	84 (32.7)	45 (33.6)	39 (31.7)
Stunting H/A, no. of children (%)			
Moderate to severe (Z-score ≤ −2)	35 (13.6)	20 (14.9)	15 (12.2)
Wasting BMI/A, no. of children (%)			
Moderate to severe (Z-score ≤ −2)	59 (23.0)	30 (22.4)	29 (23.6)
Underweight W/A^§^, no. of children (%)			
Moderate to severe (Z-score ≤ −2)*	**9** **(13.9)**	**8** **(21.6)**	**1** **(3.6)**

^#^All soil-transmitted helminth infections were of light intensity.

^§^Assessed for children under the age of 10 years; n = 65 (37 females; 28 males).

*/** Statistically significant difference between males and females (*p < 0.05; **p < 0.001).

Statistically significant differences are highlighted in bold.

**Table 2:** **Results from regression analysis highlighting significant associations between explanatories and children**’**s clinical status**, **physical fitness and cognitive capacity**

Logistic models (binary outcomes)	Association	Adjusted OR (95% CI)
*Clinical status* ^§^		
Anaemia	Age group (10–14 years)	0.50 (0.27, 0.92)
	Any form of malnutrition	1.83 (1.04, 3.21)
Stunting (HAZ-scores < −2)	Age (years)	2.34 (1.71, 3.20)
Wasting (BMIZ-scores < −2)	Any severity of stunting	2.28 (1.18, 4.40)
Any form of malnutrition (WAZ|HAZ|BMIZ-scores < −2)	Age (years)	1.21 (1.02, 1.44)
	Anaemia	1.96 (1.10, 3.47)
*Cognition* ^†^		
Low digit span test performance (LSF ≤ 4)	Grade (6)	0.42 (0.20, 0.90)
**Negative binomial model** **(count outcome)**	**Association**	**Adjusted IRR** **(95%** **CI)**
*Plasmodium* parasitaemia^§^	Haemoglobin level (g/l)	0.98 (0.97, 0.99)
**Linear/** **tobit models** **(continous outcomes)**	**Association**	**Adjusted mean difference** **(95%** **CI)**
*Physical fitness* ^§^		
VO_2_ max (ml kg^−1^ min^−1^)	Age (years)	−0.97 (−1.25, −0.70)
	Sex (female)	−2.72 (−3.70, −1.74)
	*S. mansoni* intensity (light)	1.24 (0.05, 2.43)
Hand grip strength (kg)	Age (years)	1.41 (1.01, 1.80)
	Anaemia	−1.99 (−3.30, −0.68)
	Stunting (Z-score < −2)	−4.79 (−6.70, −2.89)
Standing broad jump (cm)	Age (years)	3.45 (2.06, 4.84)
	Sex (female)	−9.95 (−14.46, −5.44)
	Wasting (Z-score < −2)	−9.82 (−14.86, −4.78)
	*S. mansoni*	6.62 (1.81, 11.43)
*Cognition* ^†^		
Code transmission test (score range: 0-20)	Grade (6)	2.73 (0.93, 4.55)
	Sex (female)	−1.49 (−2.76, −0.23)
	Wealth tertile (least poor)	1.85 (0.44, 3.27)

(+) = light intensity infection.

Reference groups of explanatories: intestinal parasites (status or intensity) = non-infected with the particular species; clinical status = not affected by particular indicator; wealth tertile = most poor, age group = 5–9 years, grade = 4^th^ grade.

^§^Fixed explanatories for adjustment in clinical and physical fitness outcomes: age, sex, socioeconomic status and anaemia or Hb.

^†^Fixed explanatories for adjustment in cognition outcomes: sex, grade and socioeconomic status. Not predetermined covariates were kept at a significance level of 0.20.

The study was carried out among 257 schoolchildren (134 females, 123 males) in December 2012 in Niablé, eastern Côte d’Ivoire.

The different types of models used according to the outcome variables are highlighted in bold.

**Figure 2:**
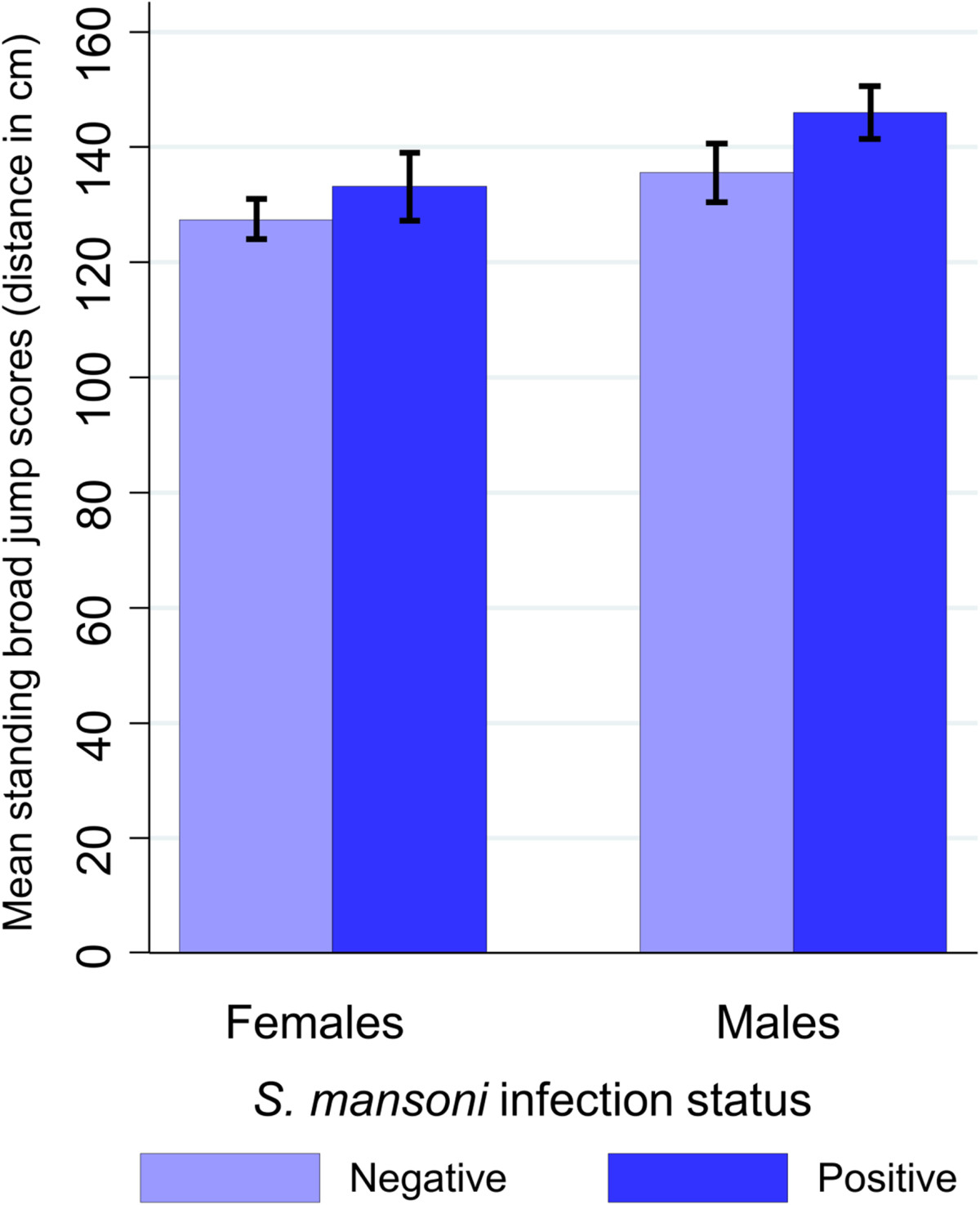
**Mean difference in standing broad jump test scores** **(distance in cm)** **for** ***S. mansoni*** **infection categories among 134 girls and 123 boys.**

Statistics from *t*-test and one-way ANOVA showed significant differences between sex, socioeconomic status and helminth infection status for specific fitness outcomes. Males had higher test scores for the standing broad jump test (141 cm *vs*. 129 cm) and for the 20 m shuttle run test (VO_2_ max: 50.6 ml kg^−1^ min^−1^*vs*. 47.7 ml kg^−1^ min^−1^) (both p < 0.001) compared to females, while no significant sex difference could be found for the grip strength test. Children from the lowest wealth tertile were found to have a significantly lower performance in the 20 m shuttle run test than children from wealthier tertiles (VO_2_ max: 48.2 ml kg^−1^ min^−1^*vs*. 49.5 ml kg^−1^ min^−1^, p < 0.05). Children with hookworm and *S. mansoni* infections were found to have higher VO_2_ max estimates and standing broad jump scores than their non-infected counterparts. These results were strongly sex confounded, since males scored better in these tests and were significantly more often infected with helminths than females. After stratification by sex, males infected with *S. mansoni* still showed a significantly better performance in the jump task than non-infected boys (Figure 2). Scores of the strength tests were positively associated with age, whilst VO_2_ max values decreased for each incremental increase of one year of age (Table 2). The positive association of *S. mansoni* light intensity and infection of any intensity remained significant with 20 m shuttle run and standing broad jump test scores, respectively, after adjusting for other covariates. Clinical morbidities, such as anaemia and malnutrition, were important predictors for physical fitness outcome measures. Particularly strength test scores seemed to be affected by malnutrition and anaemia, whilst for the 20 m shuttle run test, none of these explanatories were significant.

At baseline 117 out of 219 children (53.4%) showed low performance in the memory test, as defined by the cut-off for the LSF raw score. Low performance was higher among anaemic compared to non-anaemic children (60.7% *vs*. 47.6%, p < 0.05) and decreased with school grade (64.6%, 53.9% and 43.6% in children from grades 4, 5 and 6, respectively). After adjusting for explanatories in the regression analysis, school grade remained the only significant association for poor performance in the digit span test (Table 2). A higher school grade, wealth tertile and sex were the only significant predictors found for test performance in the code transmission task identified from multivariate tobit regression. Female sex was associated with lower attention scores, while children belonging to the highest wealth tertile performed significantly better than the most poor. Children from grade 6 achieved significantly higher scores in this test compared to 4^th^ graders. The drop-out rate for non-understanding of the test was highest among the 4^th^ grade (43.8% compared to 16.5% and 6.4% among all participants from grades 5 and 6, respectively, p < 0.001). None of the parasite infections and clinical outcomes were significantly associated with memory and sustained attention test performances in the multivariate regression analyses.

### Effect of deworming on parasitic infections, clinical status, physical fitness and cognition

The infection prevalence of *S. mansoni*, hookworm and *T. trichiura* were 8.9%, 0.9% and 0.5% at the end-of-study survey. CRs for *S. mansoni*, *S. haematobium* and soil-transmitted helminths (mainly hookworm), were 79% (60 out of 76), 100% (7 out of 7) and 91% (21 out of 23), respectively. For *S. mansoni* and hookworm, we found ERRs of 98.6% and 99.6%, respectively. Although not directly targeted by anthelminthic drugs, a clearance of all baseline positive *Entamoeba histolytica*/*E. dispar* cases (n = 9) was observed, whereas 19 out of 31 (61.3%) children with *G. intestinalis* infection at baseline were found to be free of infection at the 5-month follow-up. Both *Plasmodium* spp. infection prevalence and log-parasitaemia were significantly lower at follow-up compared to baseline (92.2% *vs*. 78.0% and 5.7 *vs* 4.1, both p < 0.001) according to Mc Nemar’s and Wilcoxon signed rank sum tests and showed similar changes in baseline helminth-infected and non-infected girls and boys (Table 3). If observation dependence was considered, however, significant differences in parasitaemia over time were observed in children with baseline helminth infections and changed anaemia status (Table 4). Children cured from hookworm infection and recovering from anaemia showed a significant decrease in *Plasmodium* parasitaemia, whilst baseline *S. mansoni*-infected children showed higher *Plasmodium* parasitaemia at follow-up.

**Table 3:** **Comparison of means for clinical**, ***Plasmodium*** **parasitaemia**, **physical and cognitive parameters in helminth**-**infected and non**-**infected schoolchildren at baseline and the 5**-**month follow**-**up surveys**, **stratified by sex**

Parameter			Baseline, mean			Follow-up, mean			Mean change (95% CI)		
	Helminth infection	N	All	Females	Males	All	Females	Males	All	Females	Males
Weight (kg)^§^	Not infected	141	31.7	32.4	30.4	33.0	33.9	31.4	1.4 (1.0, 1.8)	1.6 (1.0, 2.1)	1.0 (0.3, 1.7)
	*S. mansoni* (+)	44	31.8	33.0	31.2	33.4	34.9	32.6	1.6 (0.9, 2.2)	2.0 (1.0, 3.0)	1.4 (0.5, 2.3)
	*S. mansoni* (++/+++)	34	33.1	35.5	32.5	35.2	39.1	34.2	2.1 (1.3, 2.8)	3.6 (1.2, 6.1)*	1.7 (1.0, 2.4)
Haemoglobin (g/l)	Not infected	130	121.4	123.7	116.8	120.9	121.3	120.1	−0.5 (−3.0, 2.1)	−2.3 (−5.4, 0.7)	3.4 (−1.2, 7.9)
	Helminth^#^	89	119.0	119.7	118.8	118.6	122.1	117.3	−0.4 (−3.7, 2.8)	2.4 (−2.4, 7.2)	−1.5 (−5.6, 2.6)
*Plasmodium* parasitaemia (log (parasites/μl of blood + 1))	Not infected	129	5.5	5.4	5.7	4.0	3.9	4.2	−1.5 (−2.1, −0.9)^Δ^	−1.4 (−2.6, −0.3)^Δ^	−1.5 (−2.3, −0.7)^Δ^
	Helminth^#^	88	5.9	5.9	5.9	4.4	4.6	4.3	−1.5 (−2.3, −0.8)^Δ^	−1.6 (−2.4, −0.8)^Δ^	−1.3 (−3.1, 0.5)
VO_2_ max (ml kg^−1^ min^−1^)	Not infected	141	48.8	47.7	50.9	48.7	46.9	52.0	−0.1 (−0.8, −0.6)	−0.8 (−1.7, 0.1)	1.2 (0.0, 2.3)^†^
	*S. mansoni* (+)	44	50.3	48.1	51.3	49.4	48.0	50.0	−0.9 (−2.1, 0.3)	−0.1 (−1.8, 1.6)	−1.3 (−2.9, 0.4)*
	*S. mansoni* (++/+++)	34	49.4	47.1	50.0	51.1	45.5	52.5	1.7 (0.2, 3.2)^†^*	−1.6 (−4.9, 1.7)	2.5 (0.9, 4.1)^†^
Standing broad jump (cm)	Not infected	141	130	128	135	143	141	146	13 (10, 15)^†^	13 (10, 17)^†^	12 (8, 15)^†^
	*S. mansoni* (+)	44	140	133	144	151	140	157	11 (7, 15)^†^	7 (−0, 14)	13 (7, 18)^†^
	*S. mansoni* (++/+++)	34	146	134	149	158	148	161	12 (6, 19)^†^	13 (−2, 29)	12 (5, 19)^†^
Hand grip strength (kg)	Not infected	130	16.8	16.7	16.8	17.9	17.6	18.5	1.1 (0.4, 1.8)^†^	0.8 (−0.0, 1.7)	1.7 (0.3, 3.0)^†^
	Helminth^#^	89	17.7	17.1	18.0	18.8	17.9	19.1	1.0 (0.4, 2.0)^†^	0.8 (−1.0, 2.7)	1.1 (−0.1, 2.3)
Digit span test (score range: 2–8)^§^	Not infected	130	4.5	4.5	4.4	5.2	5.1	5.4	0.8 (0.6, 0.9)	0.7 (0.5, 0.9)	0.9 (0.6, 1.3)
	Helminth^#^	89	4.4	4.4	4.5	5.2	5.3	5.1	0.8 (0.5, 1.0)	1.0 (0.5, 1.4)	0.7 (0.3, 1.0)
Code transmission test (score range: 0–20)	Not infected	81	16.2	16.0	16.7	16.2	16.4	15.6	−0.1 (−0.8, 0.7)	0.4 (−0.5, 1.2)	−1.1 (−2.6, 0.4)
	Helminth^#^	65	15.2	12.5	16.1	15.7	15.1	15.9	0.5 (−0.4, 1.3)	2.6 (0.7, 4.5)^†^*	−0.2 (−1.1, 0.7)

(+) = light intensity infection, (++/+++) = moderate or heavy intensity infection.

^#^Infected with any soil-transmitted helminth or *Schistosoma* species of any intensity.

^§^Significant change (p < 0.05) between baseline and 5-month follow-up in all groups from paired *t*-test analysis.

^†^Significant change (p < 0.05) between baseline and 5-month follow-up for this sub-group from paired *t*-test analysis.

^Δ^Significant change (p < 0.05) between baseline and 5-month follow-up for this sub-group from Wilcoxon signed rank sum test analysis.

*Significant difference in change between infected and non-infected in this sub-group from bivariate linear regression analysis.

The study was carried out in Niablé, eastern Côte d’Ivoire between December 2012 and May 2013. Data from 219 children (112 girls, 107 boys) with complete baseline and end-of-study follow-up were considered.

**Table 4:** **Main effects** (**time**) **and significant predictors for changes over time** (**within**-**subjects effects**) **in** ***Plasmodium*** **parasitaemia**, **clinical**, **physical and cognitive outcomes after deworming from population**-**averaged GEE and random effects tobit analysis**

Model by outcome	Predictor	Change
**Logistic models** **(binary outcomes)**		**OR** **(95%** **CI)**
*Cognition* ^†^		
Change in low performance in digit span test	Time	0.27 (0.16, 0.46)*
**Negative binomial model** **(count data)**	**Predictor**	**IRR** **(95%** **CI)**
*Plasmodium* parasitaemia	Time	0.84 (0.55, 1.29)
	*S. mansoni*	6.37 (1.94, 20.88)
	Hookworm	0.19 (0.05, 0.79)
	Anaemia status (no longer anaemic)	0.18 (0.07, 0.51)
**Linear models** **(continuous outcomes)**	**Predictor**	**Coefficient** **(95%** **CI)**
*Clinical status* ^§^		
Change in Hb level (g/l)	Time	0.76 (−2.91, 4.43)
	*Plasmodium* parasitaemia (1,000+)	5.16 (0.87, 9.44)
Change in H/A Z-score (stunting)	Time	−0.19 (−0.32, −0.06)*
	Age group (10–14 years)	0.14 (0.02, 0.26)
Change in BMI/A Z-score (wasting)	Time	0.30 (−0.01, 0.60)
Weight gain (kg)	Time	0.88 (0.31, 1.45)*
	Sex (female)	0.73 (0.07, 1.39)
	*S. mansoni* intensity (++/+++)	1.06 (0.19, 1.92)
*Physical fitness* ^§^		
Change in VO_2_ max (ml kg^−1^ min^−1^)	Time	1.01 (−0.31, 2.33)
	Sex (female)	−1.40 (−2.56, −0.24)
	*S. mansoni* intensity (+)	−1.58 (−2.99, −0.16)
	Stunting severity (↑)	−2.65 (−4.52, −0.79)
Change in hand grip strength (kg)	Time	1.45 (0.71, 2.18)*
	Anaemia status (new anaemia case)	−1.59 (−3.05, −0.13)
Change in standing broad jump (cm)	Time	13.84 (10.67, 17.01)*
	Anaemia status (constantly anaemic)	−5.71 (−11.42, −0.00)
**Tobit model** **(censored count data)**	**Predictor**	**Coefficient** **(95%** **CI)**
*Cognition* ^†^		
Change in code transmission score	Time	−0.87 (−2.25, 0.50)
	Sex (female)	2.07 (0.73, 3.41)
	Helminth infection intensity (+)	2.02 (0.52, 3.52)

(+) = light intensity infection, (++/+++) = moderate or heavy intensity infection, (↑) = higher severity at follow-up.

Reference groups of explanatories: age group = 5–9 years; sex = male; intestinal parasites at baseline (status or intensity) = non-infected with a particular species; *Plasmodium* parasitaemia at baseline = parasitaemia below 1,000 parasites/μl of blood; anaemia status = constantly not anaemic; stunting severity = unchanged severity.

^§^Fixed explanatories for clinical and physical fitness outcomes: age, sex, socioeconomic status and anaemia.

†Fixed explanatories for cognition outcomes: sex, school grade and socioeconomic status. Not predetermined predictors were kept at a significance level of 0.20.

*Significant change over time (p < 0.05).

The different types of models used according to the outcome variables are highlighted in bold.

The study was carried out in Niablé, eastern Côte d’Ivoire between December 2012 and May 2013. Data from 219 children (112 girls, 107 boys) with complete baseline and end-of-study follow-up were considered.

The mean Hb levels did not differ significantly between surveys (baseline, 120.4 g/l; follow-up, 119.9 g/l; p = 0.631). The overall prevalence of anaemia remained unchanged (baseline, 34.3%; follow-up, 34.3%); the anaemia status of 88 children, however, changed. 44 became non-anaemic, while 44 children were identified as newly anaemic cases. Changes in Hb showed no significant relationship with baseline helminth infection (Table 3). Hb levels of children found with high *Plasmodium* parasitaemia at baseline showed a significant increase over time (Table 4). Overall children did not catch up in growth; on the contrary, z-scores for chronic malnutrition (i.e. stunting), further decreased compared to reference populations by −0.07 (p < 0.05). Older aged children, however, showed a significant increase in height-for-age z-scores compared to their younger peers in the population-averaged model (Table 4). On average, the children gained 1.5 kg (95% CI: 1.2, 1.8 kg) of weight over the 5-month study period. Weight gain and reduction of acute malnutrition (i.e. wasting, z-score < −2) was highest among girls, particularly those with moderate- to heavy-intensity *S. mansoni* infection at baseline (Tables 3 and 4).

**Figure 3:**
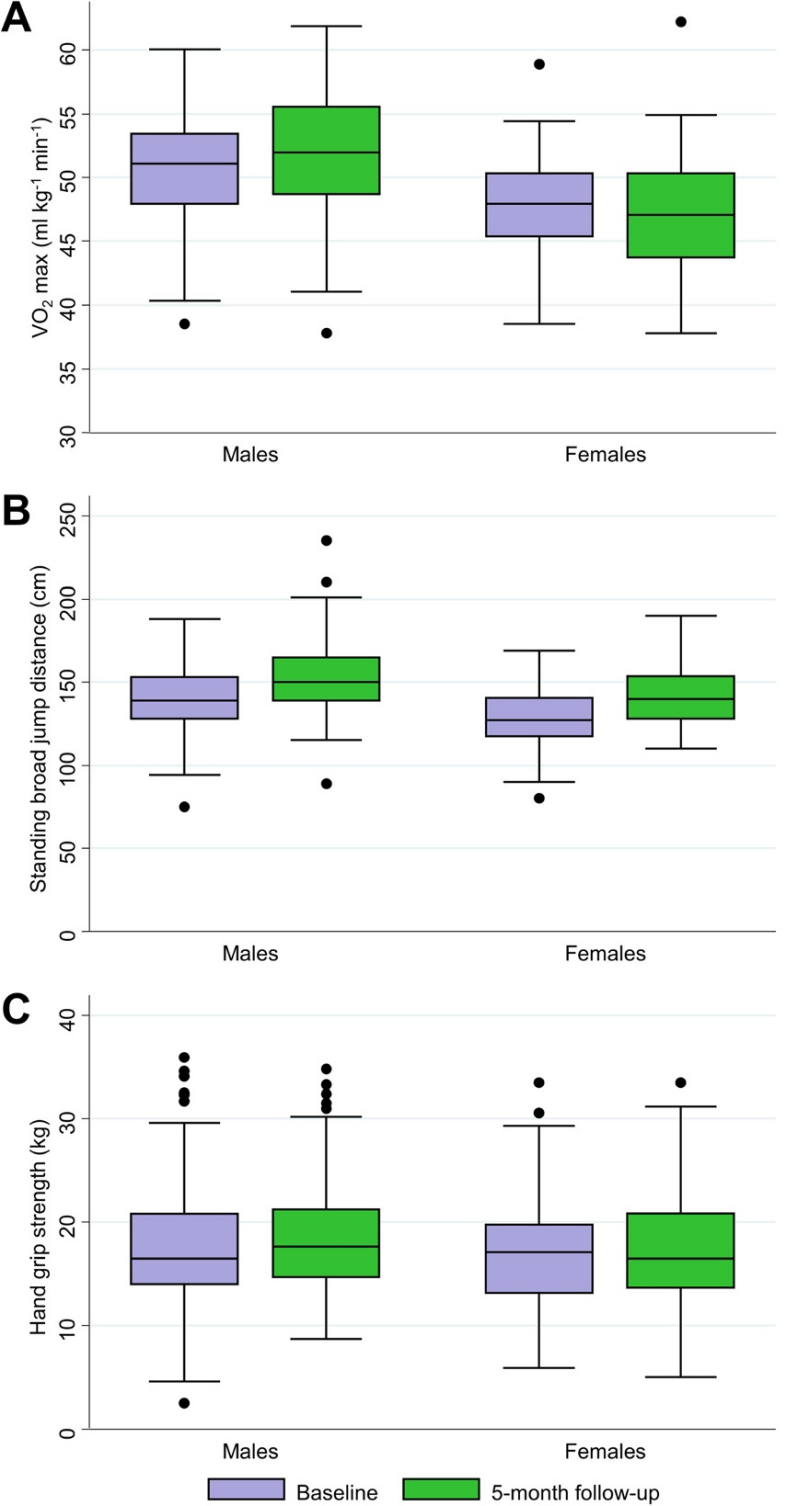
**Physical fitness performance at baseline and end**-**of**-**study survey among 219 schoolchildren**, **stratified by sex. A**: VO_2_ max estimates from the 20 m shuttle run test. **B**: Jumping distance from the standing broad jump test. **C**: Hand grip strength. Included are performances of 219 children (112 girls, 107 boys) who had complete baseline data (December 2012) and end-of-study data (May 2013). Box plot: boxes illustrate the 25^th^ and 75^th^ percentiles (ptile), while the whiskers indicate the adjacent lower and upper values (most extreme values which are within 25^th^ ptile −1.5*(75^th^-25^th^ ptile) and 75^th^ ptile + 1.5*(75^th^-25^th^ ptile), respectively). The median is shown by the line within the boxes and outliers are indicated with dots.

**Figure 4:**
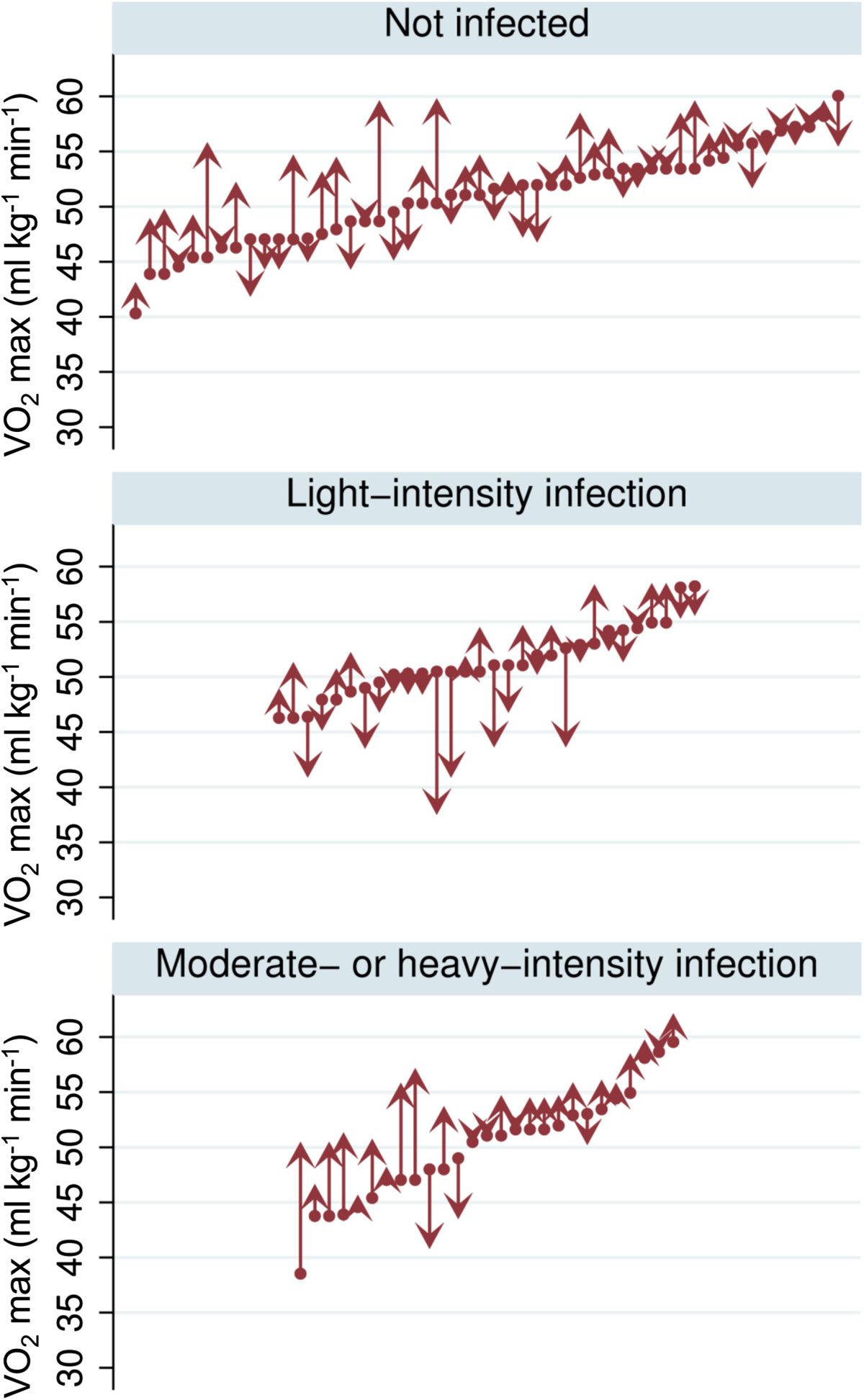
**Dynamics of VO**_**2**_ **max estimates in boys** **(n** **= 107)** **with different intensities of** ***S. mansoni*** **infection.** Upward-pointing arrows indicate improved performance at the end-of-study survey, while downward-pointing arrows stand for decreased VO_2_ max estimates at the end-of-study survey compared to baseline.

The children’s performance in the standing broad jump and the grip strength tests improved significantly over the 5-month study period with a mean increase of 12 cm (95% CI: 10, 14 cm) and 1.07 kg (95% CI: 0.49, 1.65 kg), respectively (Figure 3). The 20 m shuttle run test revealed similar VO_2_ max estimates at baseline and end-of-study surveys, but boys performed better in the follow-up assessment with a mean increase of 0.83 ml kg^−1^ min^−1^, while VO_2_ max estimates for girls decreased by −0.77 ml kg^−1^ min^−1^ (one-way ANOVA, p = 0.005). Children with moderate- to heavy-intensity *S. mansoni* infection at baseline showed a higher increase in VO_2_ max estimates at the 5-month follow-up assessment compared to their non-infected counterparts (Table 3). Especially in boys, the change in VO_2_ max estimates varied substantially between different intensities of infection. Figure 4 depicts the dynamics of VO_2_ max estimates in boys with different *S. mansoni* infection intensities at baseline. While boys without a *S. mansoni* infection and those with moderate- or heavy-intensity infection showed an improvement in aerobic capacity, lightly infected boys showed a decreasing trend with lower VO_2_ max estimates at follow-up. In girls, this effect was not observed.

Table 4 illustrates development over time and significant within-subject effects on physical fitness outcomes derived from GEE regression analyses. Status or changes of nutritional indicators remained most important predictors for changes in physical fitness outcomes. In contrast to the strength tests, significant relationships with parasitic infections and changes in VO_2_ max estimates were observed. Light-intensity *S. mansoni* infection at baseline showed a negative relationship with changes in VO_2_ max estimates.

**Figure 5:**
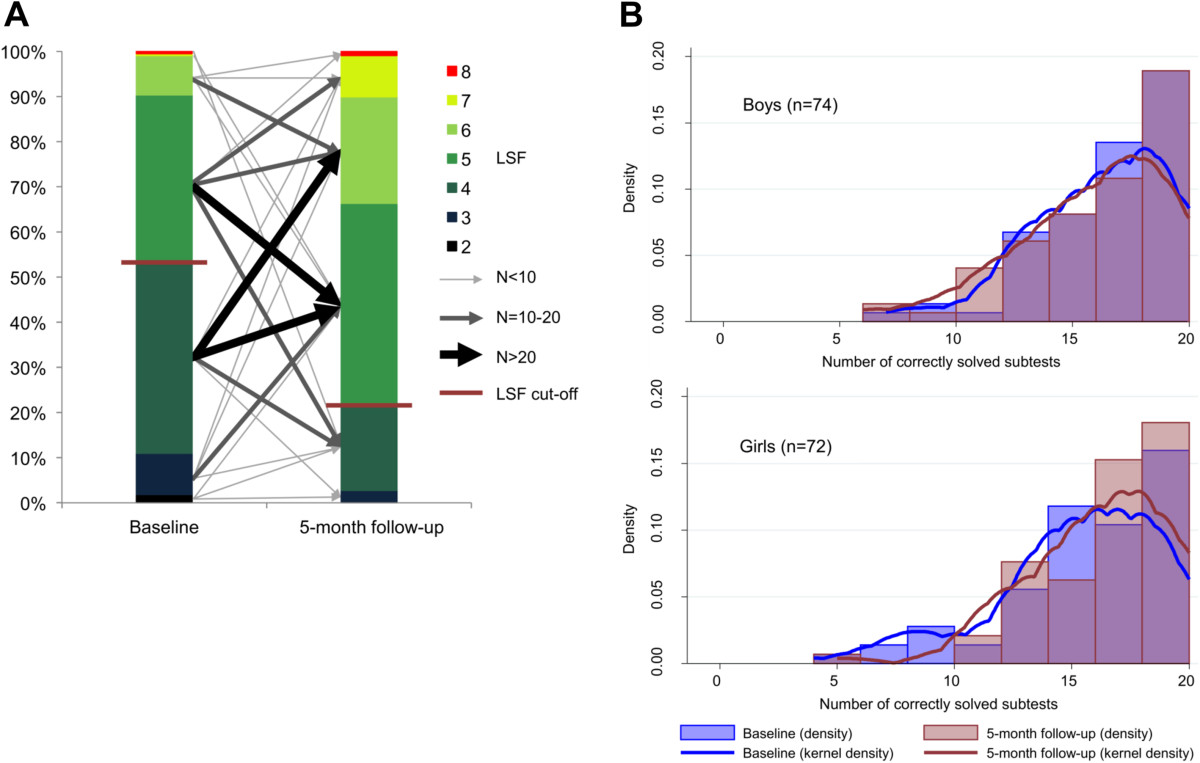
**Dynamics of cognition test scores from the forward digit span test** **(A)** **and the code transmission test** **(B).** **A**: Proportions of digit span test scores expressed as longest span forward (LSF) at baseline and end-of-study survey from 219 participants with complete data for both assessments. The arrows depict the directions of change in performance over time, whereas the width of the arrow indicates the number of children in each category of change. The cut-off to define low performance was set at LSF ≤ 4 (LSF cut-off). **B**: Relative frequency (density) of code transmission test scores (number of correctly solved subtests out of 20 subtests) at baseline and follow-up from 146 participants, stratified by sex. Girls showed a significantly higher improvement in test performance at follow-up than boys (mean difference in change between the sexes assessed by *t*-test: 1.31, p-value < 0.05).

Figure 5 illustrates the dynamics in performance for the two cognition tests. Children performed significantly better in the digit span test at the 5-month follow-up compared to baseline (p < 0.001). None of the predictors used in the basic univariate comparison and the more sophisticated GEE regression analysis showed any significant association with improved digit span test performance. Female participants performed much better in the code transmission test at follow-up compared to baseline. Furthermore, comparing for the difference in code transmission test scores between baseline and follow-up, it was found that sex and helminth infections at baseline were associated with an improvement in this test (Table 3). The random effects tobit model further revealed that, apart from female sex, particularly children with light-intensity helminth infections at baseline had a higher positive change in score at follow-up compared to their baseline non-infected counterparts (Table 4).

## Discussion

### Baseline

We present findings from an intervention study with a 5-month longitudinal follow-up among school-aged children living in a malaria-helminth co-endemic area of eastern Côte d’Ivoire. Children were subjected to a battery of parasitological, clinical, physical fitness and cognition tests at baseline and the 5-month follow-up cross-sectional surveys. The intervention consisted of two rounds of deworming (albendazole plus praziquantel) immediately after the baseline survey and 2 months later. At baseline in December 2012, we found that 91.1% of the children harboured *P. falciparum* parasites in their blood. As we only collected a single finger-prick blood sample, the ‘true’ *Plasmodium* infection rate might reach 100%. Using duplicate Kato-Katz thick smears from a single stool sample, we found *S. mansoni* and hookworm prevalences of 35.4% and 9.7%, respectively. Interestingly, boys had much higher helminth prevalences than girls (*S. mansoni*, 53.7% *vs*. 18.7%; hookworm, 18.7% *vs*. 1.5%). These observations are somewhat in line with findings from a recent national cross-sectional school-based survey conducted between November 2011 and February 2012 in Côte d’Ivoire, as boys showed significantly higher prevalences than girls, but not as marked as in the current study [25]. Given the very high *Plasmodium* infection rate, our findings focusing on helminth infections should thus be interpreted as *Plasmodium*-helminth co-infections.

About one third of the children surveyed had clinical manifestations, most importantly anaemia (34.6%) and moderate to severe malnutrition (32.7%). These clinical parameters were negatively associated with children’s physical fitness, as determined by three standard tests [37, 38]. Helminth infections, on the other hand, showed no clear association with clinical outcomes. Hence, our study highlights the importance of a deeper mechanistic understanding of how helminths and other parasites mediate pathways to ill-health and impaired physical fitness [11, 23, 46]. Our findings are in agreement with a recent study assessing physical fitness in Kenyan schoolchildren, which identified nutritional deficiencies as major predictors for impaired physical fitness performance, whilst there was no robust association with parasitic diseases [13]. These observations might also explain why the VO_2_ max estimates from the current and a previous study in Côte d’Ivoire [14] were comparable with findings from the Kenyan study, where the extent of malnutrition and anaemia was similar. In contrast, a cohort of school-aged children in Yunnan province, People’s Republic of China, had a strikingly high rate of stunting and helminth infections [15, 21], and VO_2_ max estimates and strength test scores were substantially lower than in age-matched African children [13, 14]. Although we were not able to identify clear relationships between parasitic infections and baseline performance in physical fitness and cognition tests, possible interactions between intestinal schistosomiasis and malaria should be considered. Indeed, boys concurrently infected with *Plasmodium* and *S. mansoni* showed significantly higher broad jump scores and in the multivariate regression analysis, VO_2_ max estimates were positively associated with light-intensity *S. mansoni* infection. Anaemia was found to be associated with high *Plasmodium* parasite densities and negatively impacted on fitness outcomes. Our results from the longitudinal models revealed lower *Plasmodium* parasitaemia in *S. mansoni*-infected children (data not shown) and thus may indicate an indirect beneficial effect on clinical and physical condition from co-infection. However, it remains to be determined whether this positive association is related to a potential protective effect of chronic schistosomiasis on malaria pathology, as suggested by different groups [47, 48], or whether causality goes the other way around. It is also conceivable that more physically fit children show higher activity patterns, which include water-contact activities, and thus are at higher risk of schistosomiasis [49].

### Effects of deworming

The deworming with two rounds of albendazole (400 mg) and praziquantel (40 mg/kg) spaced by 2 months was highly efficacious against soil-transmitted helminths, while 16 children (21.1%) continued to excrete *S. mansoni* egg in their stool, although at drastically reduced egg loads. *Plasmodium* spp. prevalence was lower at the 5-month follow-up than baseline. Malaria incidence depends on rainfall patterns. Considering 2-week intervals before the baseline (December 2012) and follow-up surveys (May 2013) in our study area, rainfall patterns were comparable [29]. The clearance of helminth infections and the much reduced intensities among those who remained helminth-positive showed conflicting effects on *Plasmodium* parasitaemia depending on the respective helminth species. While hookworm-infected children showed reduced IRRs for parasitaemia at follow-up, the opposite was found in children infected with *S. mansoni*. Enhancing effects on antimalarial immune responses favoured by schistosomiasis co-infection and reduced *Plasmodium* parasitaemia in individuals with light-intensity *Schistosoma* infections in other West African co-endemic settings have been highlighted in previous studies [50–52]. Similarly to our findings with regard to hookworm, repeated anthelminthic treatment positively impacted on *Plasmodium* infection and parasitaemia among *Ascaris*-infected children in Nigeria [53].

Given the lack of measurable benefit of deworming on anaemia, Hb levels and stunting over the 5-month observation period, clinical morbidity is likely to be malaria-driven or induced by other non-investigated issues (e.g. other infectious diseases, non-communicable diseases, nutritional deficiencies and genetic Hb disorders) [54]. We found a significant weight gain among children within the 5-month study period, which conforms to the weight gain in a healthy reference population of similar age range [55]. We identified a higher weight gain in girls compared to boys, and in moderate- to heavy-intensity *S. mansoni*-infected individuals. Several other studies showed benefits on growth catch-up after deworming in chronic schistosomiasis [56, 57]. Yet, interpretation of effects on growth can be complex considering that there are two types of catch-up growth. First, recovery of a chronic disease after an intervention. Second, catch-up growth is linked to hormonal changes, also referred to as growth spurt in puberty, which usually starts earlier in girls [56, 58].

We observed substantial improvement in the two strength tests after deworming. Since both test outcomes were positively associated with age, we would expect better performance over time; to identify potential benefits from deworming we thus have to compare between baseline helminth infected, which were cured from infection or showed considerably reduced egg output at follow-up, respectively, *vs*. baseline non-infected children. For the standing broad jump test, where we found significantly better performances in *S. mansoni*-infected individuals at baseline, no difference in change of test achievement between infected and non-infected individuals was observed at the 5-month treatment follow-up. It is conceivable that 5 months is too short of a time period to observe any measurable change in explosive leg power after deworming in a malaria hyper-endemic area. In contrast, changes in VO_2_ max estimates after deworming showed an interesting pattern in *S. mansoni*-infected children. Children with heavy infections seemed to benefit from deworming, while children with light-intensity infections showed a significant decrease. This finding underlines the importance of considering helminth infection intensity when determining potential effects from *Plasmodium*-helminth interactions. Previous studies highlighted possible protective effects from light-intensity helminth infections on clinical consequences in malaria co-endemic settings [19, 47, 59]. Unanticipated negative health effects from deworming campaigns in areas where malaria co-exists are conceivable and must be investigated in the future [60, 61].

Working memory, as assessed by a digit span test, improved dramatically in our child cohort. Nonetheless, we were unable to directly attribute this improvement to the deworming intervention, since we found no significant relationship between helminth infection and test outcomes neither in the baseline survey nor considering changes over the 5-month study period. In attention scores, however, we observed a significant increase at follow-up for individuals with baseline helminth infections (*Schistosoma* or soil-transmitted helminths) and substantially decreased worm loads and cleared infection at follow-up, respectively. Ezeamama and colleagues also found significantly increased cognition scores after praziquantel treatment against *S. japonicum* infection in participants free of infection during a 12-month follow-up period [62]. There is a paucity of high-quality studies investigating the effect of deworming on children’s cognitive ability. Moreover, we lack setting-specific information regarding the effect of *Plasmodium* infection on children’s cognition, which may have altered potential benefit from deworming. In a highly malarious area in Uganda, a significant negative association between *P. falciparum* parasitaemia and code transmission test scores was found in schoolchildren [17]. A randomised controlled trial of intermittent preventive treatment (IPT) against malaria showed improved cognition test results in Kenyan children [63].

### Strengths and limitations

We consider our results from the physical and cognitive tests as valid, as we adhered to standard protocols [64–66]. Importantly, the tests are sufficiently flexible to allow for local adaptions, so that they can be implemented in resource-constrained settings. Although external influences (i.e. climate, test ground) could not be completely excluded, the physical fitness outcomes showed expected relationships with age and sex, as documented in previous studies in developed countries [38, 67, 68], with the exception of sex differences in grip strength. Regarding the latter issue, the same observation was made in rural Kenyan pupils [69]. It is conceivable that children’s daily activities in rural Africa involve physical exertion, as part of livelihood strategies, which are shared by both sexes [70]. The two selected cognition tests were comprehensible. For future studies, however, we would recommend the code transmission task to be done with children attending grade 5 or above or to replace it with a more appropriate test in lower grades, as we had considerable drop-outs in 4^th^ graders. Cognition results were coherent with regard to the link between a better performance and a higher education grade [70]. Both cognitive and physical functioning does not only depend on capacity, but also on factors such as concentration, mood and motivation on the day of the tests [71, 72]. Ideally, several rounds of testing should be performed for each child, with averages taken for subsequent analysis. Due to time constraints and in order to minimise disruptions of day-to-day activities in school, repeated testing was not feasible. Efforts were made to enhance test-retest reliability and to minimise effects due to repeated testing for physical and cognitive ability by a number of familiarising procedures (e.g. careful explanations, exercise sessions and warm-up tasks).

We administered deworming drugs twice keeping children worm-free or achieve low worm loads until the end-of-study follow-up, since reinfection has been shown to damp potential beneficial effects from deworming on physical fitness [21, 62]. We did not consider different treatment groups, which would have allowed comparison between differences in children who received drugs and placebo, respectively. To which extent drug administration contributed to improvements in physical fitness and cognition is therefore not straightforward. As most children were infected with *Plasmodium,* the evaluation of *Plasmodium*-related effects on outcomes is impossible due to the lack of a ‘healthy’ reference group. We addressed this point by discriminating between different levels of parasitaemia. A further limitation of our study is the relatively short follow-up period (5 months). Some beneficial effects might only become observable if interventions continue over a longer period and follow-up assessments at a later stage are considered. More robustness and more clear trends in cognitive ability could be achieved by administering a more comprehensive battery of learning, memory and attention domains as done for previous studies looking at schistosomiasis and school performance [62, 73]. Our study sample was above the calculated minimum number of participants needed to show effects of clinical relevance in physical fitness, but sex-specific helminth prevalence differed and thus group sizes were not equal among males and females. Relationships between helminth infection and test outcomes which are associated with sex (e.g. VO_2_ max, standing broad jump distance) may therefore lack power. However, effects on VO_2_ max estimates of males still maintain a power of 80% at an alpha error of 5% considering that the ratio of helminth-infected *vs*. non-infected males was roughly 1:1.

## Conclusions

Taken together, our findings show limited effects of deworming on helminth-related morbidity, physical functioning and cognitive ability in school-aged children in a highly malaria-endemic setting of Côte d’Ivoire. Hence, the potential benefit of deworming in hyper-endemic malaria settings with stable transmission are likely to be tempered by overshadowing consequences due to *P. falciparum* infection, though reduced *Plasmodium* parasitaemia after deworming was observed. Nevertheless, we reported negative associations between helminth infection and physical and cognitive ability among those children who had growth and haematological deficits, which include as underlying causes helminth infections. The conflicting findings and potential beneficial effects from light-intensity helminth infections identified urge for a deeper mechanistic understanding of *Plasmodium*-helminth interactions and how such interactions influence disease-related morbidity. Our study could not identify major detrimental effects on *Plasmodium*-related pathology in helminth co-infected individuals, a potential increase of *Plasmodium* parasitaemia in *S. mansoni*-infected children, however, was indicated after two rounds of deworming. For future intervention studies assessing effects on clinical and subtle morbidity in helminth-malaria co-endemic settings different interventions and study designs could be an option to consider. These may include the use of supplementary treatment besides deworming, which also impact on *Plasmodium* spp. infection and directly on clinical outcomes (e.g. IPT or administration of iron-fortified food products), implemented as a longitudinal cohort study with a follow-up period of at least 2 years. We regard the measures used to assess physical and cognitive impairment as appropriate, but future studies may profit from a repeated measures approach and a prolonged follow-up period to strengthen findings and to assess not only potential short-term effects. With regard to control and morbidity reduction, our findings highlight the need of combined strategies for achieving greater impact and forestalling potential exacerbating effects in settings with stable malaria transmission.

## Electronic supplementary material

Additional file 1: Detailed procedures of physical fitness testing.(DOCX 22 KB)

Below are the links to the authors’ original submitted files for images.

Authors’ original file for figure 1

Authors’ original file for figure 2

Authors’ original file for figure 3

Authors’ original file for figure 4

Authors’ original file for figure 5

## Abbreviations

AIC: Akaike information criterion
BMI: Body mass index
CI: Confidence interval
CR: Cure rate
DALY: Disability-adjusted life year
ERR: Egg reduction rate
GEE: Generalised estimating equation
Hb: Haemoglobin
IPT: Intermittent preventive treatment
IRR: Incidence rate ratio
LSF: Longest span forward
NTD: Neglected tropical disease
OR: Odds ratio
QIC: Quasi-likelihood information criterion
RDT: Rapid diagnostic test
SAF: Sodium acetate-acetic acid-formalin
SSSU: Service de Santé Scolaire et Universitaire
STH: Soil-transmitted helminth
TEA-Ch: Tests of Everyday Attention for Children
WISC-IV: Wechsler Intelligence Scale for Children - Fourth Edition.
