# Supplementary material for: Effect of deworming on school-aged children’s physical fitness, cognition and clinical parameters in a malaria-helminth co-endemic area of Côte d’Ivoire
Source: BMC Infect Dis. 2014 Jul 25;14:411. doi: 10.1186/1471-2334-14-411 (PMC4131038; doi:10.1186/1471-2334-14-411)
Supplement: Supplementary file 1 — Additional file 1: Detailed procedures of physical fitness testing.(DOCX 22 KB) [file 12879_2014_3726_MOESM1_ESM.docx]

**Additional File 1. Detailed procedures of physical fitness testing.**

The standing broad jump test was implemented according to adapted standard operating procedures (SOPs) from a study on a school-based physical activity programme (KISS) conducted in Switzerland [1]. In brief, the distance (in cm, precision 1 cm) from the take-off line to the point, where the back of the heel nearest to the take-off line landed on the ground, after jumping with both feet together was measured. The test was repeated until two valid test scores could be achieved, whereof the better then was recorded.

Hand grip strength (in kg, precision of 0.5 kg) was assessed using a digital hand grip dynamometer (T.K.K. 5401 Grip-D; Takey, Tokyo, Japan). For each participant the dynamometer was adjusted for optimal grip span values proposed by Espana-Romero et al. [2] after measurement of hand span (with a precision of 0.5 cm). For this test as well the higher score of two attempts was recorded.

For the conduction of the 20 m shuttle run test [3] children were asked to run in groups of maximal 10 individuals forth and back on a 20 meter course, which had been marked with two lines on the ground. The test started with an initial running pace of 8.0 km/h and a progressive 0.5 km/min raise of the running speed given by a beep signal from a software run on a portable computer (Team beep test software; Bitworks Design, Cheltenham, UK). To assure that all children run with equal speed and according to the pace given by the sound signal they were accompanied by a field assistant. Scores, expressed as current stage and number of laps of the current stage, were recorded for each child, who ended the test either by giving up or by no longer being able to follow the pace and not reaching the 20 m line two consecutive times.

**References:**

1. Kriemler S, Zahner L, Schindler C, Meyer U, Hartmann T, Hebestreit H, Brunner-La Rocca HP, van Mechelen W, Puder JJ: **Effect of school based physical activity programme (KISS) on fitness and adiposity in primary schoolchildren: cluster randomised controlled trial.** *BMJ* 2010, **340**:c785.

2. España-Romero V, Artero EG, Santaliestra-Pasias AM, Gutierrez A, Castillo MJ, Ruiz JR: **Hand span influences optimal grip span in boys and girls aged 6 to 12 years.** *J Hand Surg Am* 2008, **33**:378–84.

3. Yap P, Fürst T, Müller I, Kriemler S, Utzinger J, Steinmann P: **Determining soil-transmitted helminth infection status and physical fitness of school-aged children.** *J Vis Exp* 2012:e3966.
