# Supplementary figures and images for: Effect of deworming on school-aged children’s physical fitness, cognition and clinical parameters in a malaria-helminth co-endemic area of Côte d’Ivoire
Source: BMC Infect Dis. 2014 Jul 25;14:411. doi: 10.1186/1471-2334-14-411 (PMC4131038; doi:10.1186/1471-2334-14-411)

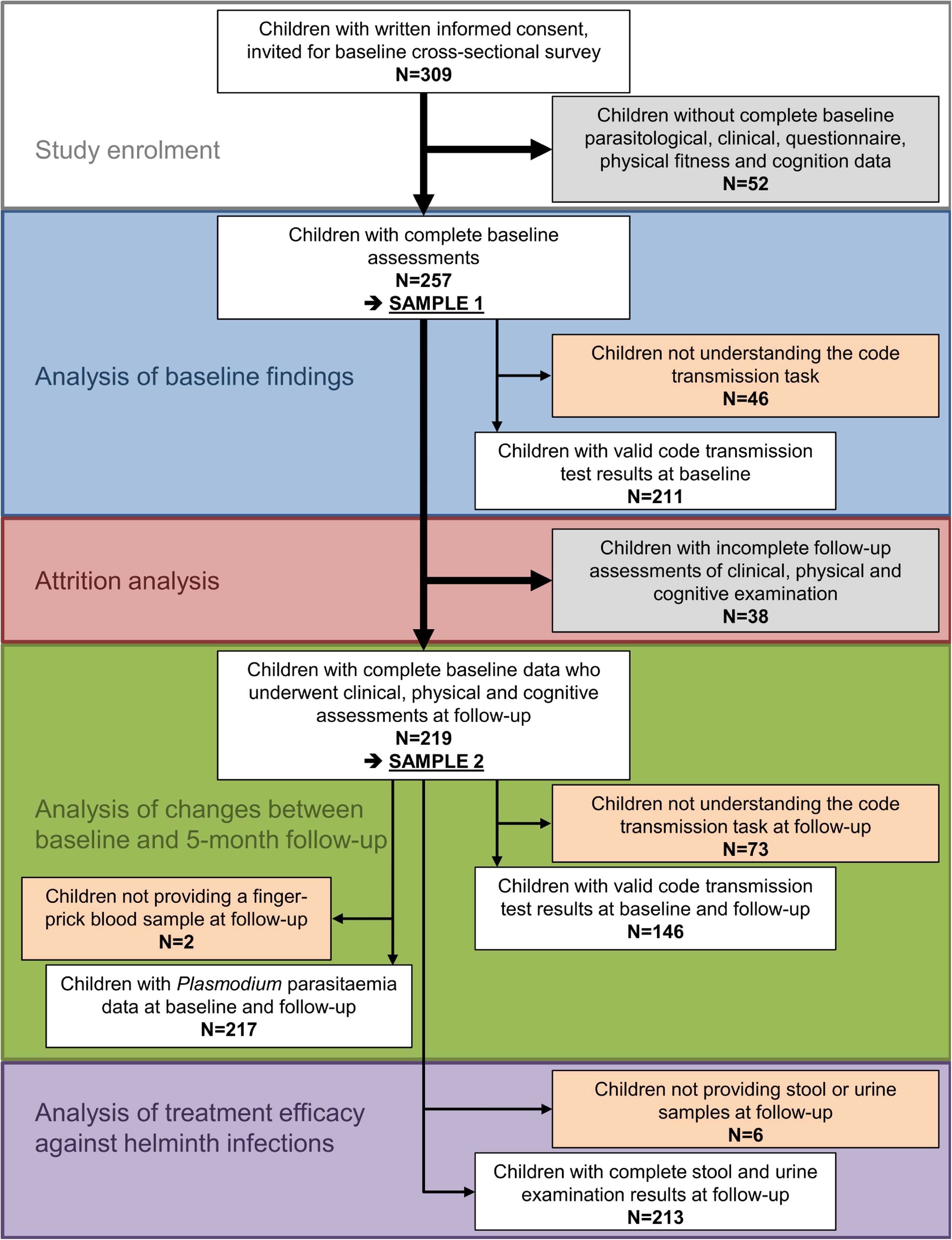

Supplement: Supplementary file 2 — Authors’ original file for figure 1 [file 12879_2014_3726_MOESM2_ESM.tiff]

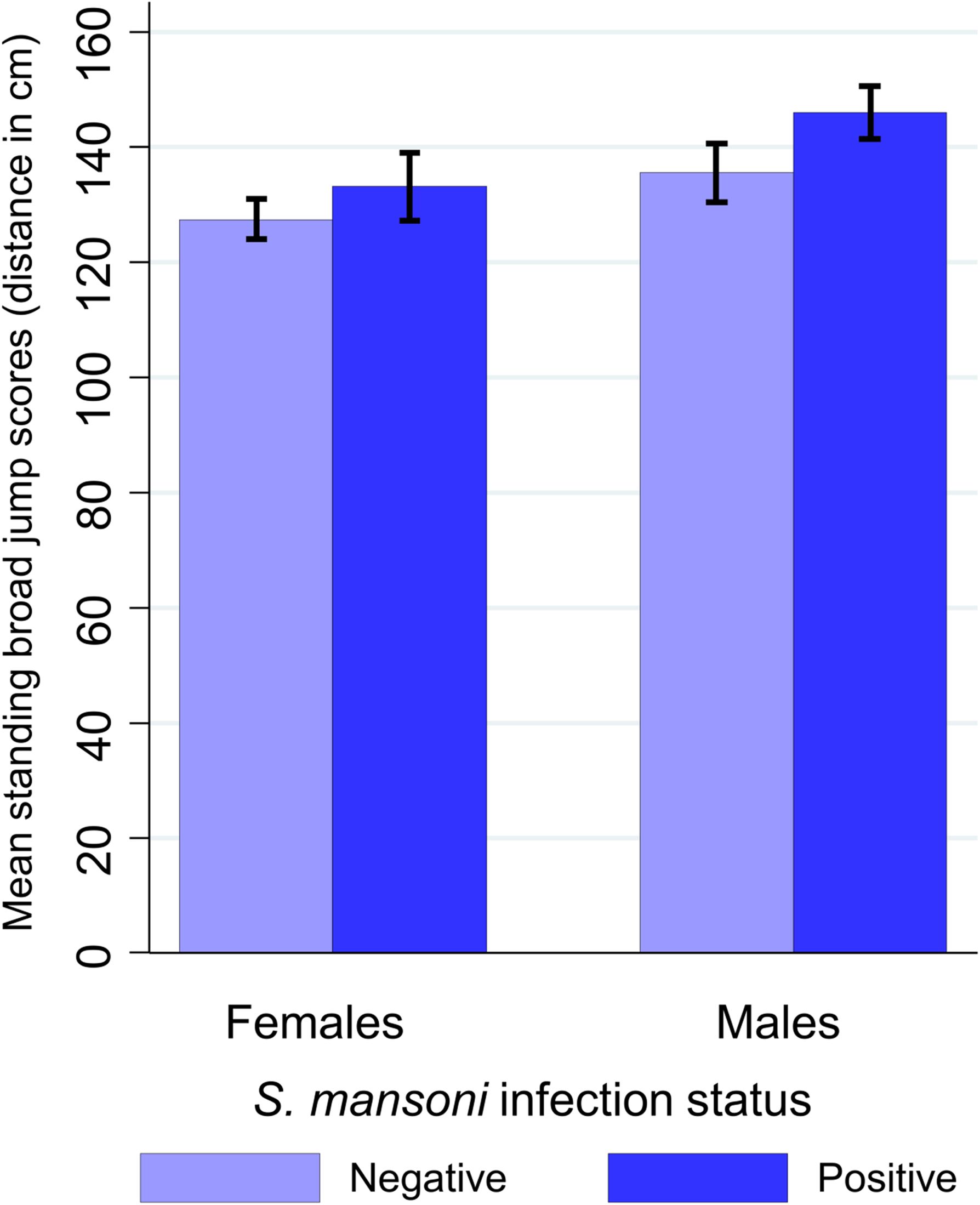

Supplement: Supplementary file 3 — Authors’ original file for figure 2 [file 12879_2014_3726_MOESM3_ESM.tif]

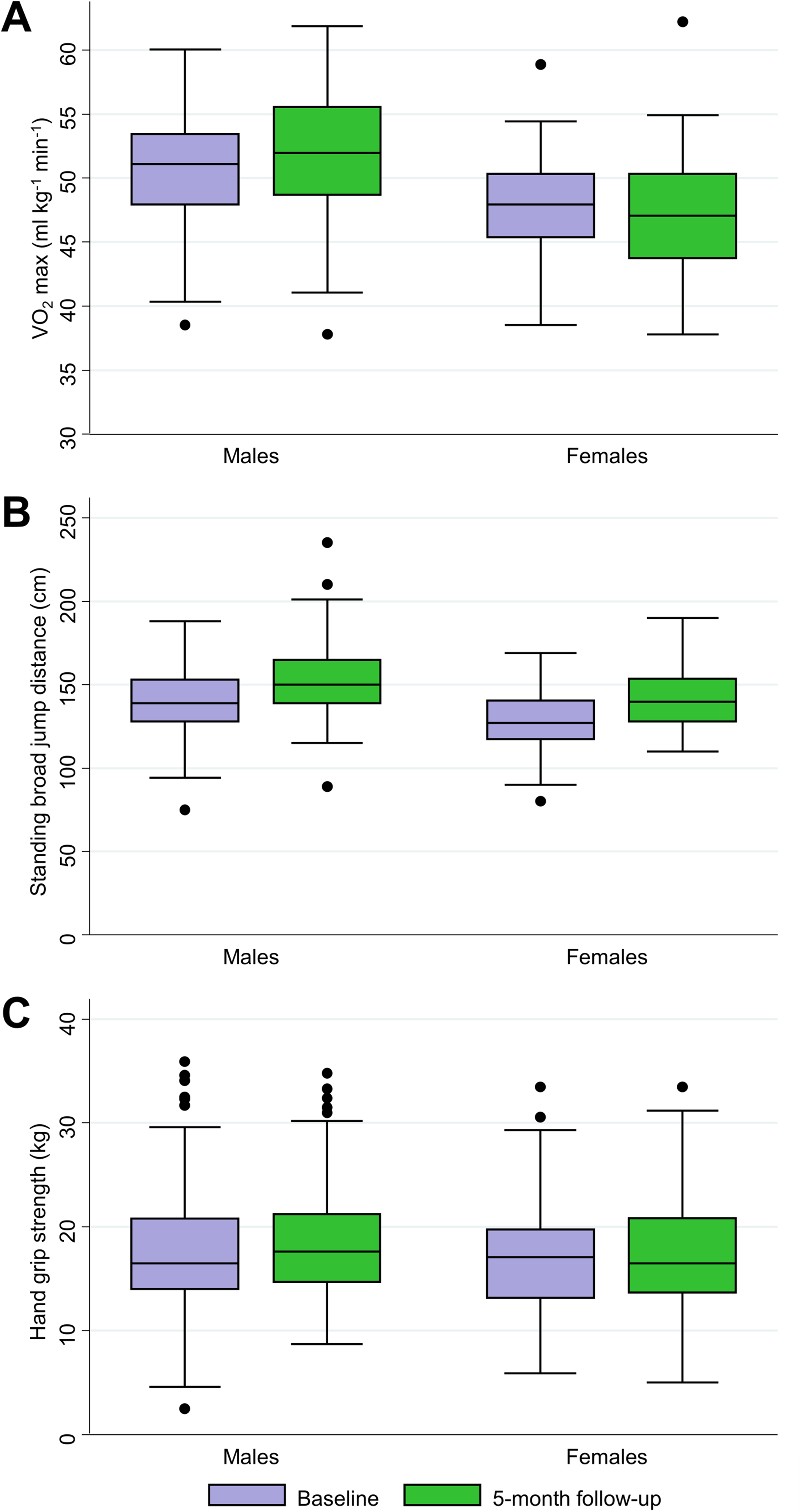

Supplement: Supplementary file 4 — Authors’ original file for figure 3 [file 12879_2014_3726_MOESM4_ESM.tif]

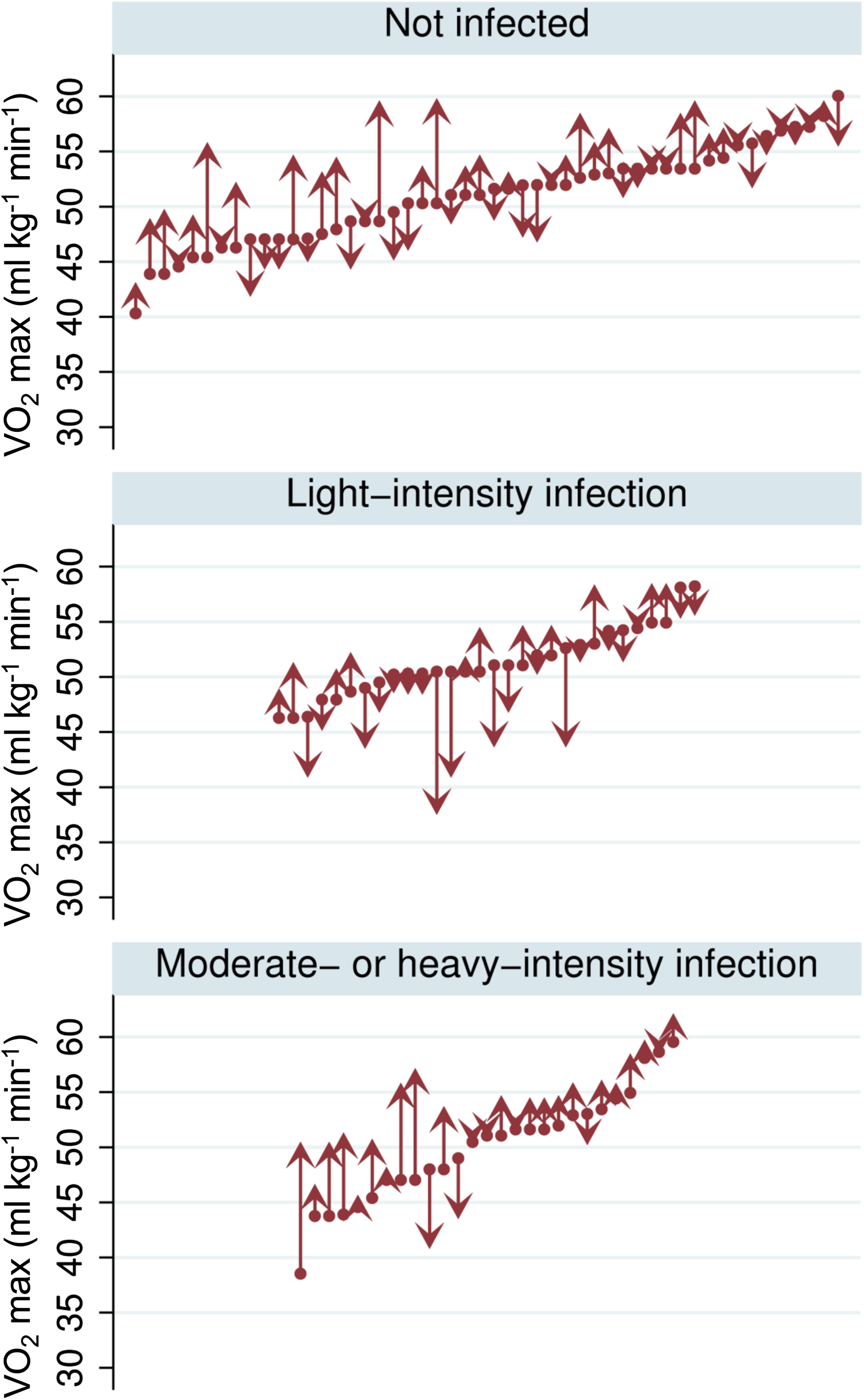

Supplement: Supplementary file 5 — Authors’ original file for figure 4 [file 12879_2014_3726_MOESM5_ESM.tif]

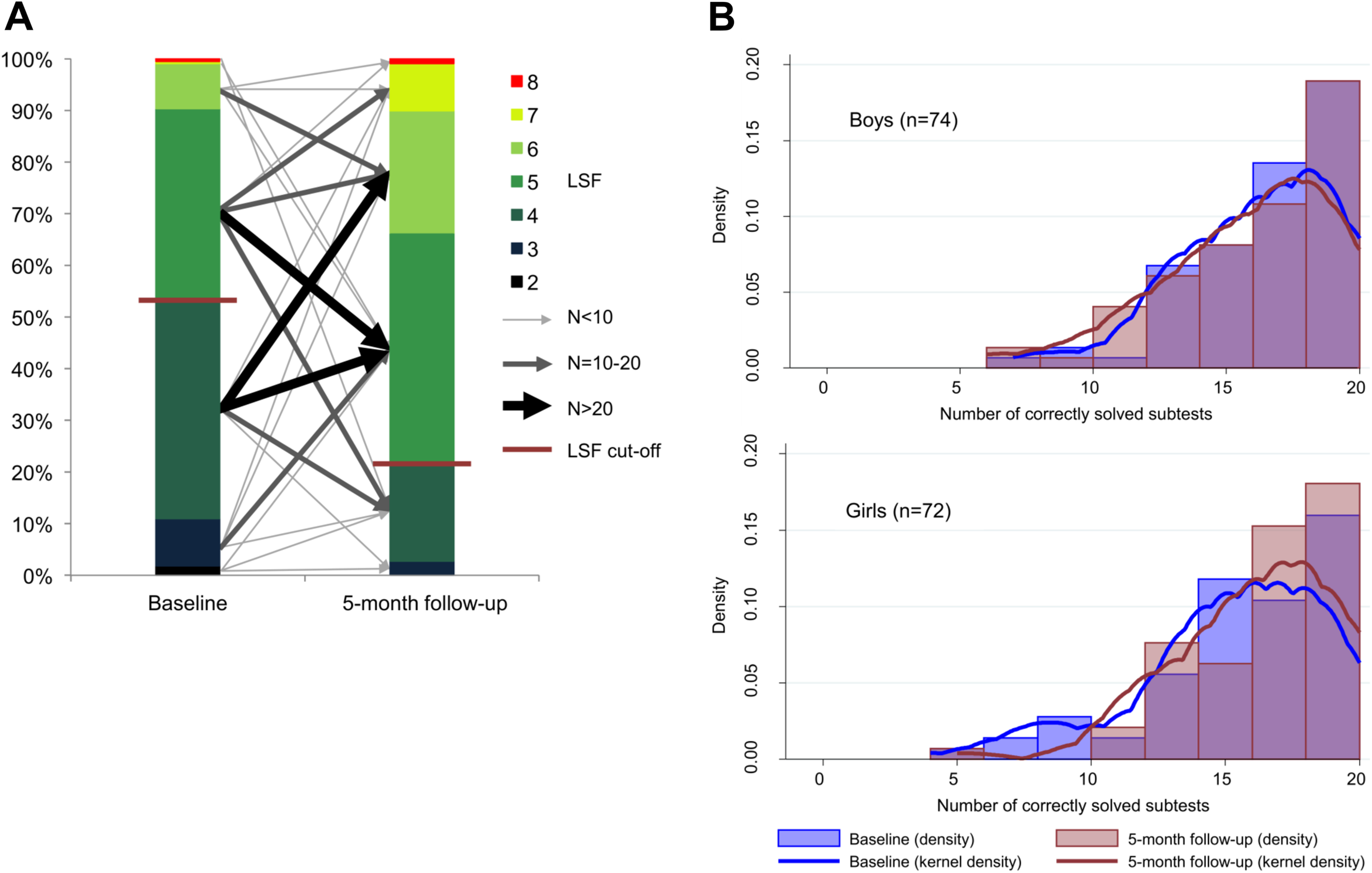

Supplement: Supplementary file 6 — Authors’ original file for figure 5 [file 12879_2014_3726_MOESM6_ESM.tif]
